# Supplementary material for: Rapid emergence of climate change in environmental drivers of marine ecosystems
Source: Nat Commun. 2017 Mar 7;8:14682. doi: 10.1038/ncomms14682 (PMC5343512; doi:10.1038/ncomms14682)
Supplement: Supplementary Information — Supplementary Notes, Supplementary Table, Supplementary Figures and Supplementary References [file ncomms14682-s1.pdf]

Supplementary Note 1

Trend in ecosystem drivers

For the 'business-as-usual' scenario, the annual maximum in SST increases by 0.31 °C per decade (global median) with intensified warming in the tropics and northern hemisphere high latitudes (Supplementary Fig. 8). This rate is slightly larger than that of the annual mean SST trend (0.26 °C per decade; Supplementary Fig. 2), indicating that summer peak temperatures are increasing more rapidly than the mean. In both metrics, the slowest warming occurs in regions of deep water formation in the Southern Ocean and subpolar North Atlantic. The annual minimum (and mean) pH decreases fairly uniformly by -0.25 % per decade, with a more pronounced decline in the Arctic and subpolar North Atlantic. High latitude regions are currently undersaturated in CO_2 with respect to the atmosphere partly due to the restriction of air-sea gas exchange by sea ice [1]. Retreating ice results in deeper mixing and freshening of surface layers, thus accelerating absorption of atmospheric CO_2 [2,3]. Regionally, the annual minimum in PP and interior oxygen content exhibit both decreasing and increasing trends, although globally there is a decreasing trend of -1.2 % per decade (annual mean -0.8 % per decade) for PP and -0.75 % per decade (annual mean -0.6 % per decade) for oxygen. The climate change trend in PP is largest in the North Atlantic, reaching -4 % per decade (for annual mean). Whilst the annual mean PP decreases, the annual minimum value increases (i.e. winter PP values increase over time). The collapse of the phytoplankton bloom in this region, which is currently strongly seasonal, reflects a shift toward nutrient limited conditions in response to increased stratification [4]. Climate change has a positive effect on PP only at very high latitudes (Supplementary Fig. 2; poleward of 60 °S and 70 °N), particularly in regions where retreating sea ice exposes new open water habitat and leads to an extended ice-free growing season [5,6]. The seasonal minimum oxygen content of thermocline waters generally decreases by -0.8 % per decade (annual mean -0.5 % per decade) in mid latitudes and by -2 % per decade (annual mean similar) in high latitudes, reflecting the reduced solubility of gases in warm waters and a weaker resupply of oxygen to the ocean interior due to enhanced stratification. Counterintuitively, interior oxygen content shows a positive response to ocean warming in low latitude upwelling regions, increasing by up to 3 % per decade (annual mean 2 % per decade). This is consistent with paleoproxy data [7] which suggests that reduced upwelling, due to increased stratification, results in reduced PP and export of organic matter into the ocean interior, and thus a reduction in oxygen demand, which outweighs the decrease in oxygen arising from reduced solubility and mixing.

In the mitigation scenario, the spatial patterns of the trends in both the annual extrema and the annual means are similar (Supplementary Fig. 2 and 8), but the magnitude is smaller. Global median trend for the annual maximum SST is 0.14 °C per decade (annual mean 0.12 °C per decade), annual minimum pH is -0.15 % per decade (annual mean -0.14 % per decade), annual minimum PP is -0.81 % per decade (annual mean -0.41 % per decade), and annual minimum interior oxygen content is -0.43 % per decade (annual mean -0.35 % per decade).

Supplementary Note 2

ToE estimated using annual means, rather than annual extrema

Previous work on emergence of climate change signals has tended to use annual mean model output to define trends and emergence. Here we argue that the relevant metric for ecosystem adaptation is the seasonal maxima/minima, as organisms must already be well-adapted to at least the range in seasonal variability. Although the climate change trend is more rapid for annual extrema than for the annual mean (compare Supplementary Fig. 2 and Fig. 8), the natural variability is also larger so that the ToE is delayed and the pace of climate change is slower (compare Fig. 1 and Supplementary Fig. 3). Mitigation has a similar effect on ToE and pace for the annual means as for the annual extrema, delaying emergence by ~ 20 years and slowing the pace of climate change by ~ 15-30 years (Supplementary Fig. 4). This general pattern is borne out in the maps of multiple driver combinations which demonstrate that climate change trends more rapidly exceed the bounds of natural variability when the annual mean is considered, rather than the annual extrema (compare Fig. 2 and Supplementary Fig. 5). The overall effect on emergence of drivers of ecosystem stress is displayed in Supplementary Fig. 6, where multiple stress emerges in 73 % of the ocean in the next 15 years in the 'business-as-usual' scenario (52 % for mitigation scenario) and in 95 % of the ocean by 2050 (85 % for mitigation scenario). By the end of the century, climate change trends in all 4 ecosystem drivers have emerged in 78 % of the ocean (56 % for mitigation scenario).

Supplementary note 3

Robustness of model results

Model fields are compared to observations using Taylor diagrams [8] in Supplementary Fig. 10, both for the annual mean and annual extrema. Modelled SST corresponds very closely to observations with correlation coefficients > 0.95 for all models for both annual mean and annual extrema. Interior oxygen is also well represented by the majority of models

(correlation coefficient > 0.8), with the exception of the annual mean in the CNRM and MPI-MR models. PP and pH are less well simulated by the models, although PP is derived from an empirical algorithm applied to satellite data so has large uncertainty itself. In particular, because modelled pH is very spatially uniform compared to the observations, correlation is weak (generally 0.1-0.5). For PP, the annual minimum value is better modelled (correlation coefficient of 0.4-0.7) than the annual mean. Despite the acknowledged deficiencies in some aspects of the CMIP5 models, they have been successfully used in many climate change studies, e.g. [9,10].

Stippling on Supplementary Fig. 8 indicates low model agreement based on robustness measures suggested in [11]. High robustness for SST and pH is defined as when the model-mean difference (between 2100 and 2006) in a property exceeds the inter-model standard deviation. For interior dissolved oxygen and PP, high robustness is defined as when at least 80 % of the models agree on the sign of the trend, again following [11]. The probability densities of the trends for SST, PP, pH and interior oxygen (Supplementary Fig. 9) are all unimodal, as tested using the Dip test of unimodality [12] with a 5 % critical level. This demonstrates overall agreement on the global trends between the models. The inter-model variability (expressed as interquartile range) in the ToE is low (< 10 years) for the majority of the ocean for pH, larger for SST (~ 20 years), and largest for PP and interior oxygen (~30-40 years). The predicted ToE is therefore most robust for pH and SST and correspondingly lower for PP and oxygen.

Supplementary Table 1: List of models from the CMIP5 multi-model ensemble archive (http://cmip-pcmdi.llnl.gov/cmip5/data_portal.html) used in this study. Asterisks next to model names indicate that additional monthly resolution oxygen concentration output was made available.

Modeling Center (or Group)	Institute ID	Model Name	Reference
Community Earth System Model Contributors	NSF-DOE-NCAR	CESM1(BGC)*	[13]
Centre National de Recherches Météorologiques / Centre Européen de Recherche et Formation Avancée en Calcul Scientifique	CNRM-CERFACS	CNRM-ESM1*	[14]
NOAA Geophysical Fluid Dynamics Laboratory	NOAA GFDL	GFDL-ESM2G*<br>GFDL-ESM2M*	[15,16]
Met Office Hadley Centre (additional HadGEM2-ES realizations contributed by Instituto Nacional de Pesquisas Espaciais)	MOHC (additional realizations by INPE)	HadGEM2-CC<br>HadGEM2-ES	[17]
Institut Pierre-Simon Laplace	IPSL	IPSL-CM5A-LR*<br>IPSL-CM5A-MR*<br>IPSL-CM5B-LR	[18]
Max-Planck-Institut für Meteorologie (Max Planck Institute for Meteorology)	MPI-M	MPI-ESM-MR*<br>MPI-ESM-LR*	[19]
Norwegian Climate Centre	NCC	NorESM1-ME*	[20]

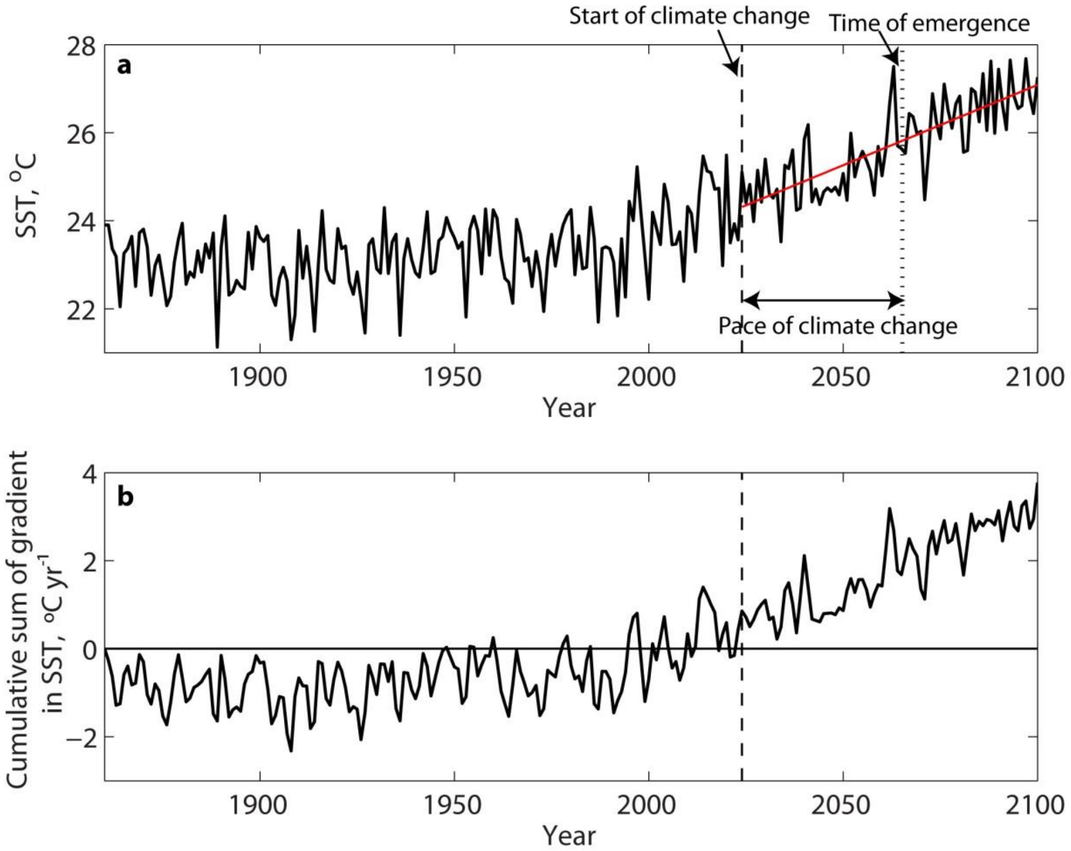

Supplementary Figure 1: Example time series. Example of estimated metrics in SST for 34 °N, 130 °W from the GFDL-ESM2M model showing a) time series of SST for conjoined historical and warming (RCP8.5) runs with the inflection point ('start of climate change signal') marked with dashed line and the time of emergence marked with dotted line. The trend is calculated between these 2 years (red line). The 'pace of climate change' is the time between the inflection point and ToE. b) The inflection point is calculated as the year when the cumulative sum of the gradient in SST exceeds zero for the remainder of the time series. See Methods for more details.

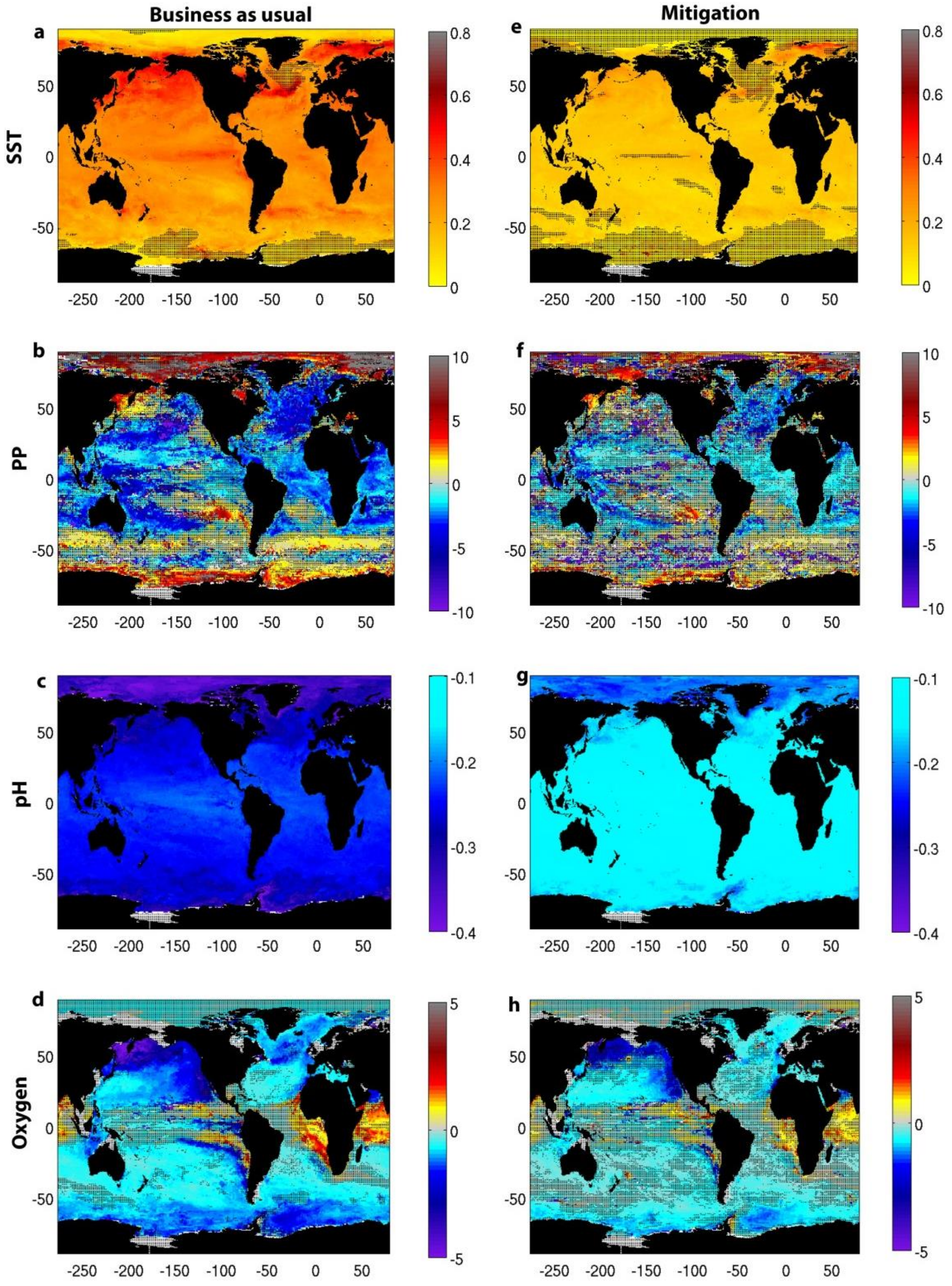

Supplementary Figure 2: Trend in annual mean for ecosystem drivers. Trend in a multi-model mean ensemble of CMIP5 output run under a 'business-as-usual' scenario (RCP8.5) and a mitigation scenario (RCP4.5). a) Sea surface temperature (°C per decade); b) PP, c) pH and d) interior oxygen content, all expressed as % change per decade with respect to the mean of 1986-2005. Panels e-h) same, but for the mitigation scenario (RCP4.5). White areas indicate where the regression is not statistically significant ($p > 0.05$). Stippling indicates where inter-model agreement is low (see Methods).

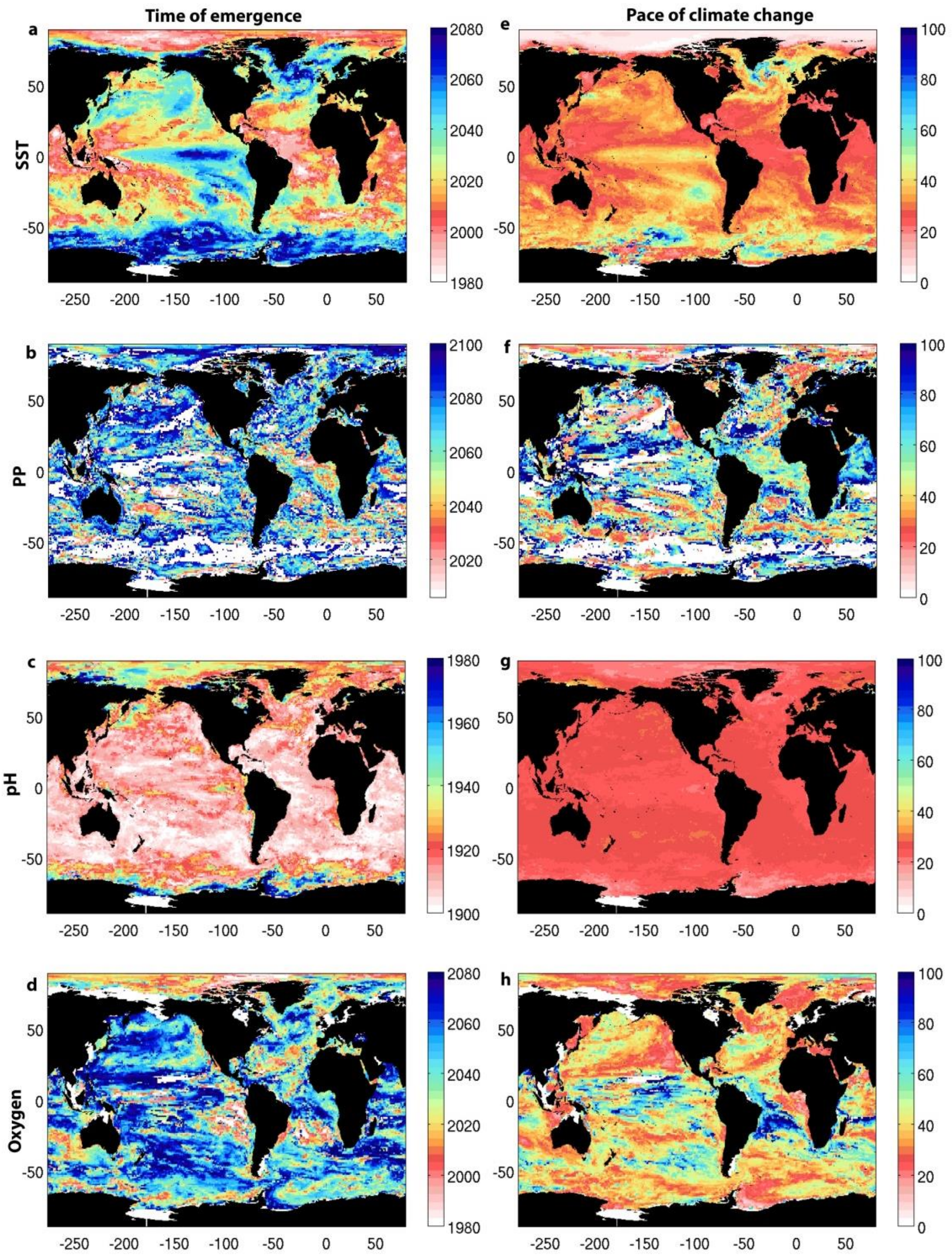

Supplementary Figure 3: Time of emergence and pace of climate change in ecosystem drivers for annual mean model output. Multi-model median of year when climate change trend exceeds the range of natural interannual variability for a) sea surface temperature, b) primary production, c) pH and d) interior oxygen content in the 'business-as-usual' scenario (RCP8.5). Note the different colour scales for each variable. Panels e)-h), number of years between the start of climate change and the signal emerging for annual mean model output (see Methods). White areas indicate where ecosystem drivers do not emerge above the range of interannual variability for that parameter by 2100.

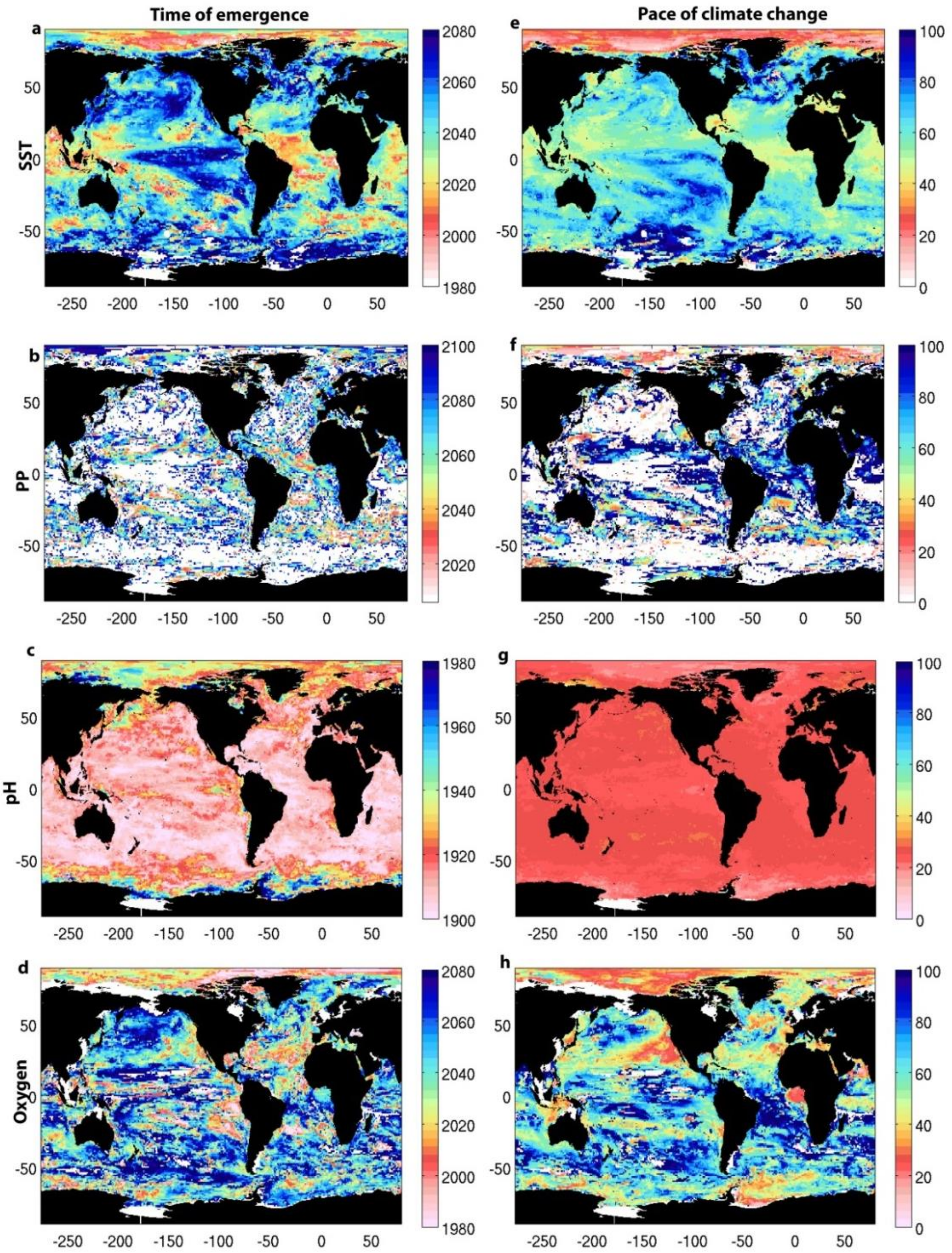

Supplementary Figure 4: Time of emergence and pace of climate change in ecosystem drivers for annual mean model output under a mitigation scenario. Multi-model median of year when climate change trend exceeds the range of natural interannual variability for a) sea surface temperature, b) primary production, c) pH and d) interior oxygen content in a mitigation scenario (RCP4.5). Note the different colour scales for each variable. Panels e)-h), number of years between the start of climate change and the signal emerging for annual mean model output (see Methods). White areas indicate where ecosystem drivers do not emerge above the range of interannual variability for that parameter by 2100.

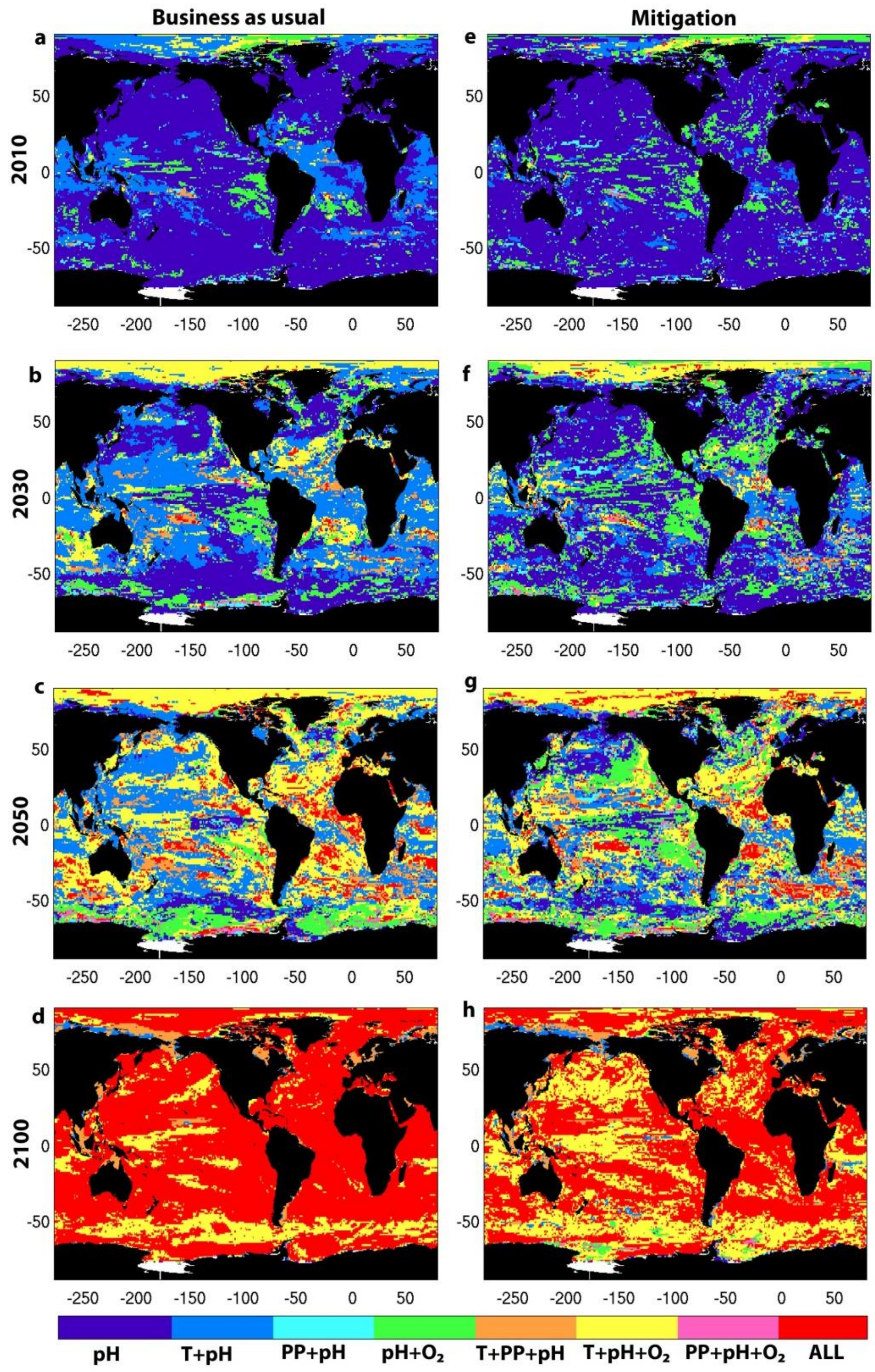

Supplementary Figure 5: Emergence of multiple ecosystem drivers in annual mean model output. Combination of stressors that have emerged above the background of interannual variability by a) 2010, b) 2030, c) 2050 and d) 2100 for a 'business-as-usual' scenario (RCP8.5). Panels e)-h) same, but for the mitigation scenario (RCP4.5). In legend, T refers to sea surface temperature, PP to primary production and O_2 to interior oxygen concentration.

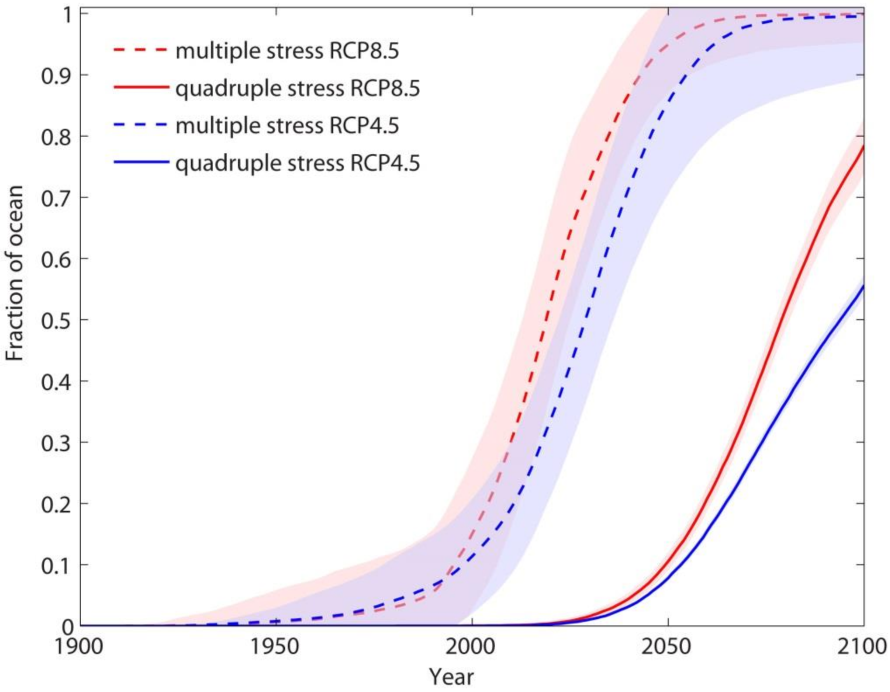

Supplementary Figure 6: Effect of mitigation on the global emergence of drivers of ecosystem stress in annual mean model output. The proportion of the ocean in each year 1900-2100 affected by multiple stress (> 1 driver) and quadruple stress (all 4 drivers) in the 'business-as-usual' scenario (RCP8.5) and a mitigation scenario (RCP4.5). Shaded areas represent the inter-model standard deviation.

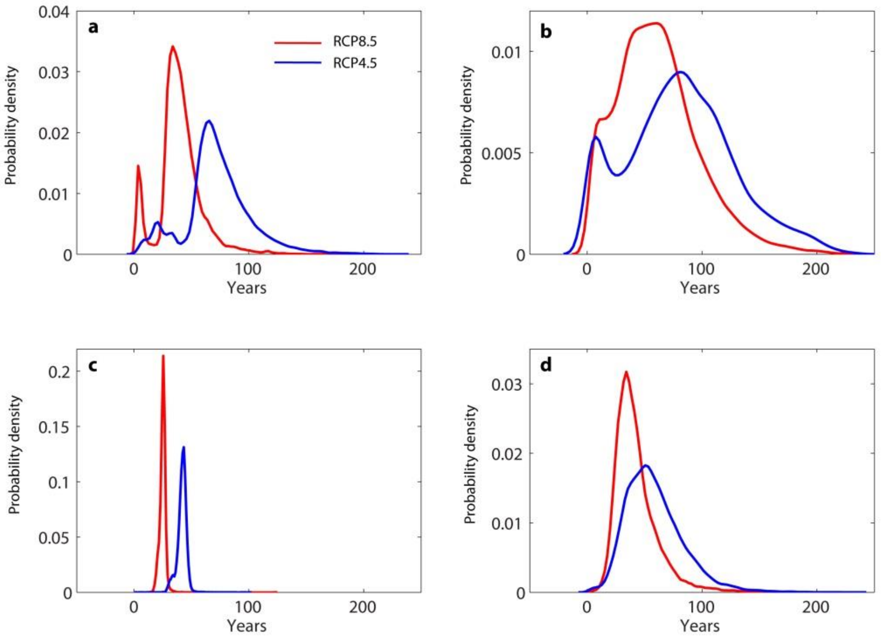

Supplementary Figure 7: Probability density plots of the pace of climate change in annual extrema (in years) in the RCP8.5 (red line) and RCP4.5 (blue line) scenarios for a) SST, b) PP, c) pH and d) interior dissolved oxygen. Median (and interquartile range) for RCP8.5 are 38(±17), 58(±46), 25(±3) and 38(±20) years for SST, PP, pH and interior oxygen, respectively. Median (and interquartile range) for RCP4.5 are 70(±28), 80(±62), 42(±4) and 55(±31) years for SST, PP, pH and interior oxygen, respectively.

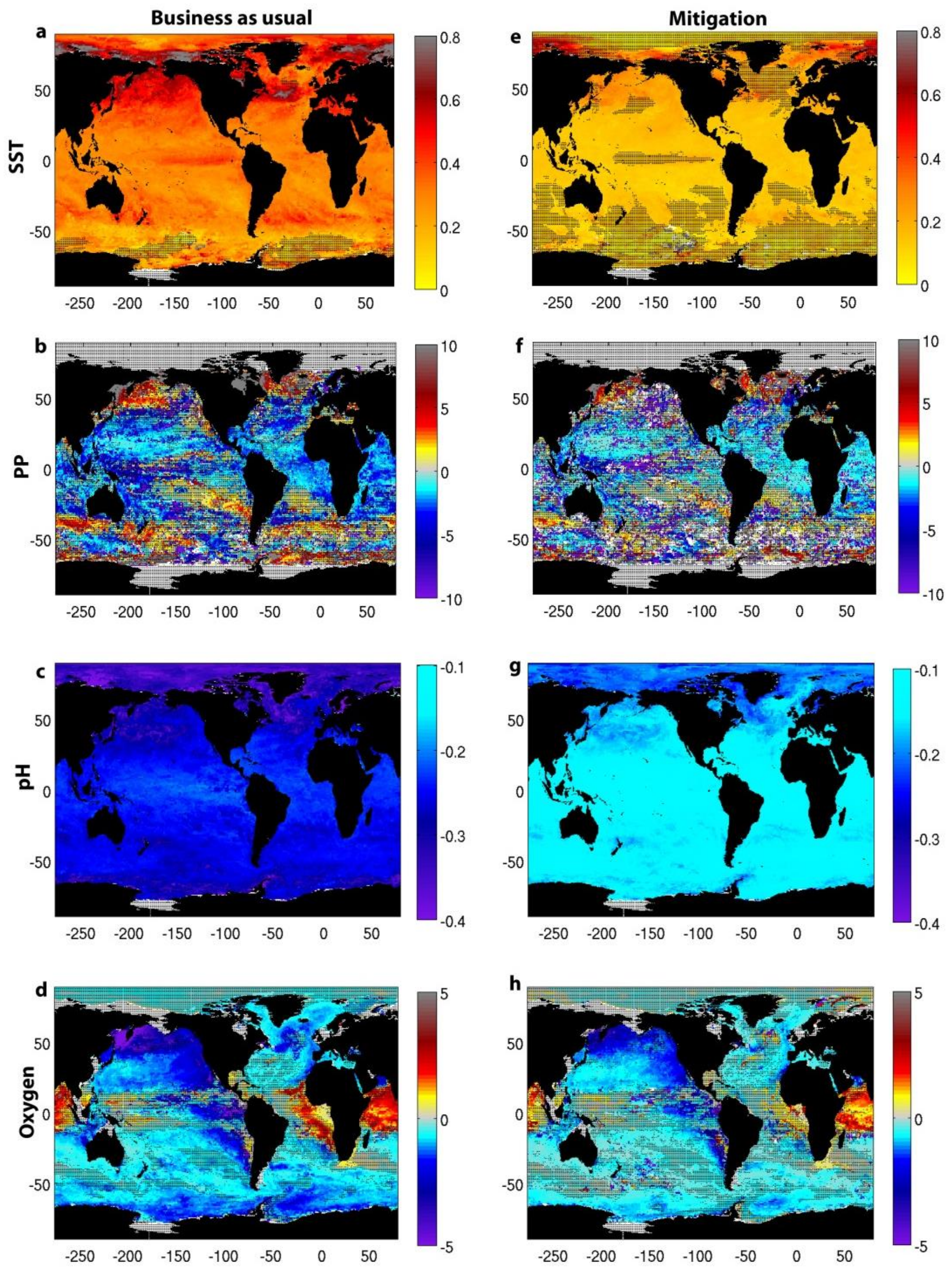

Supplementary Figure 8: Trend in annual extrema of ecosystem drivers. Trend in a multi-model mean ensemble of CMIP5 output run under a 'business-as-usual' scenario (RCP8.5) and a mitigation scenario (RCP4.5). a) Annual maximum in sea surface temperature (°C per decade); annual minimum in b) PP, c) pH and d) interior oxygen content, all expressed as % change per decade with respect to the mean of 1986-2005. Panels e-h) same, but for the mitigation scenario (RCP4.5). White areas indicate where the regression is not statistically significant ($p > 0.05$). Stippling indicates where inter-model agreement is low (see Methods).

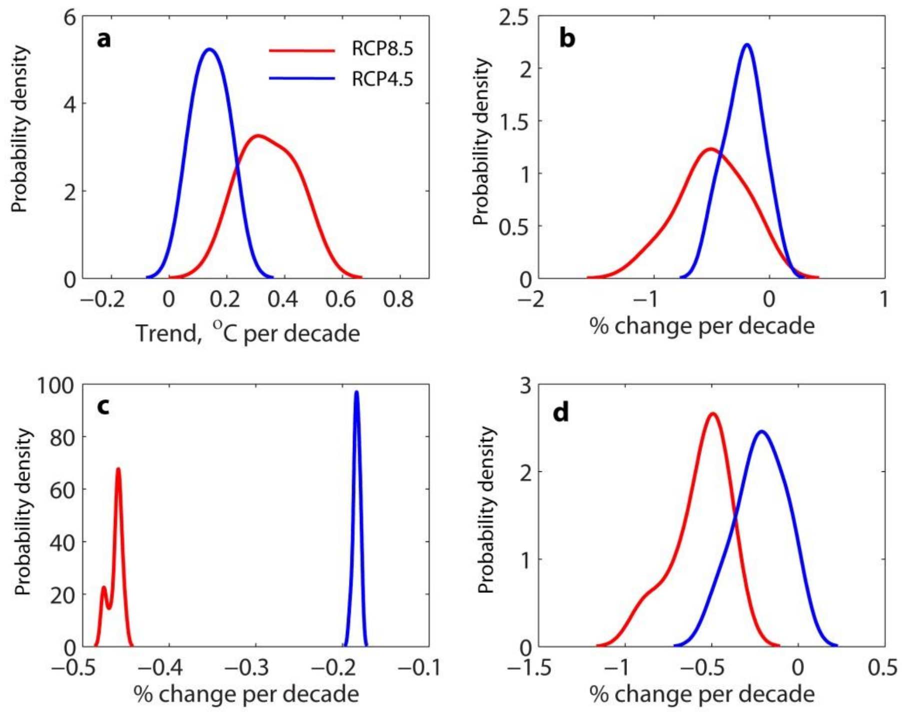

Supplementary Figure 9: Probability density plots of the global trend in annual extrema for 2006-2100 in the RCP8.5 (red line) and RCP4.5 (blue line) scenarios for a) SST, expressed as °C per decade, b) PP, c) pH and d) interior dissolved oxygen, expressed as % change per decade with respect to 1986-2005. Median (and interquartile range) for RCP8.5 are 0.31(±0.15), -0.52(±0.35), -0.45(±9 x 10^{-3}) and -0.53(±0.17) for SST, PP, pH and interior oxygen, respectively. Median (and interquartile range) for RCP4.5 are 0.13(±0.05), -0.20(±0.2), -0.18(±5 x 10^{-3}) and -0.20(±0.2) for SST, PP, pH and interior oxygen, respectively.

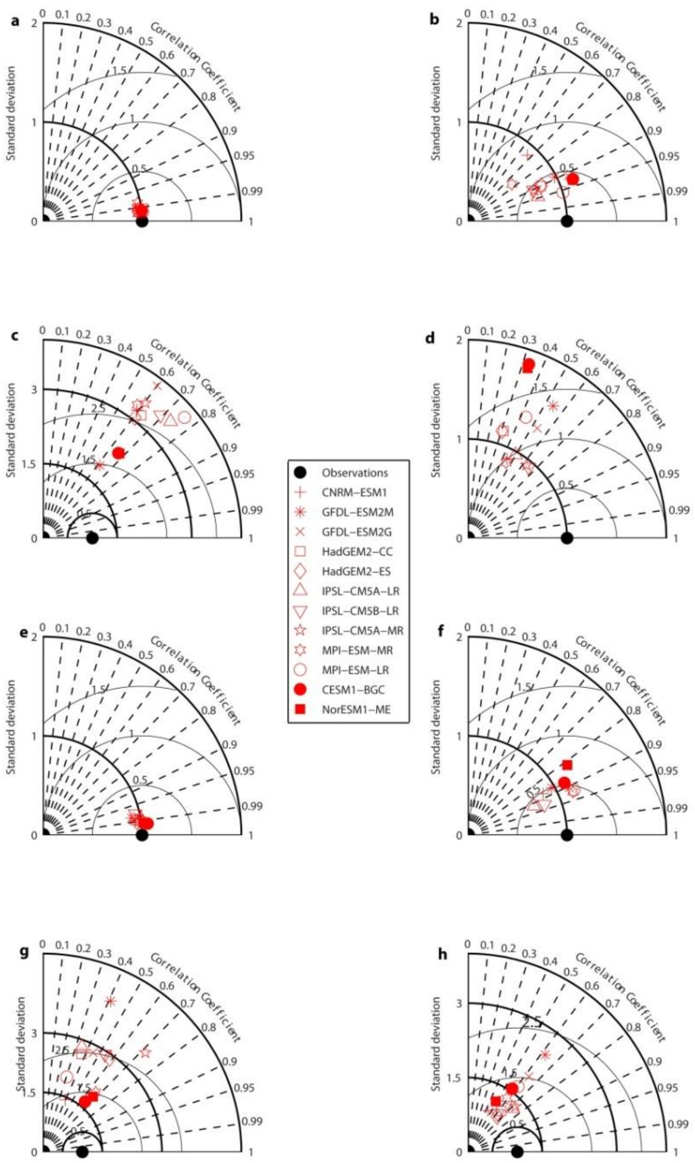

Supplementary Figure 10: Taylor diagrams representing correspondence between observations and models. Each model is compared to observed mean a) SST, b) interior oxygen content , c) pH and d) PP, and observed annual extrema in e) SST, f) interior oxygen content and g) pH and h) PP. The angular coordinates show the correlation coefficient (R), radial coordinates show the normalised standard deviation (model/observations) and semi-circles show the centred root mean square difference between model and observations [8]. A model that perfectly matched observations would coincide with the black dot. Observations are from the World Ocean Atlas 2013 [21,22] climatology for oxygen and temperature, satellite-derived PP calculated using the Vertically Generalised Production Model [23] where coastal points (water depth < 500 m) have been removed, and pH from the Takahashi climatology [24].

Supplementary References

1 McNeil, B. I., Tilbrook, B. & Matear, R. J. Accumulation and uptake of anthropogenic CO2 in the Southern Ocean, south of Australia between 1968 and 1996. *Journal of Geophysical Research-Oceans* **106**, 31431-31445, doi:10.1029/2000jc000331 (2001).

2 McNeil, B. I., Metzl, N., Key, R. M., Matear, R. J. & Corbiere, A. An empirical estimate of the Southern Ocean air-sea CO2 flux. *Global Biogeochemical Cycles* **21**, GB3011, doi:10.1029/2007gb002991 (2007).

3 Popova, E. E., Yool, A., Aksenov, Y., Coward, A. C. & Anderson, T. R. Regional variability of acidification in the Arctic: a sea of contrasts. *Biogeosciences* **11**, 293-308, doi:10.5194/bg-11-293-2014 (2014).

4 Henson, S., Cole, H., Beaulieu, C. & Yool, A. The impact of global warming on seasonality of ocean primary production. *Biogeosciences* **10**, 4357-4369, doi:10.5194/bg-10-4357-2013 (2013).

5 Arrigo, K. R., van Dijken, G. & Pabi, S. Impact of a shrinking Arctic ice cover on marine primary production. *Geophysical Research Letters* **35**, L19603, doi:10.1029/2008gl035028 (2008).

6 Vancoppenolle, M. *et al.* Future Arctic Ocean primary productivity from CMIP5 simulations: Uncertain outcome, but consistent mechanisms. *Global Biogeochemical Cycles* **27**, 605-619, doi:10.1002/gbc.20055 (2013).

7 Deutsch, C. *et al.* Centennial changes in North Pacific anoxia linked to tropical trade winds. *Science* **345**, 665-668, doi:10.1126/science.1252332 (2014).

8 Taylor, K. E. Summarizing multiple aspects of model performance in a single diagram. *Journal of Geophysical Research - Atmospheres* **106**, 7183-7192 (2001).

9 Mora, C. *et al.* The projected timing of climate departure from recent variability. *Nature* **502**, 183-187, doi:10.1038/nature12540 (2013).

10 Bopp, L. *et al.* Multiple stressors of ocean ecosystems in the 21st century: projections with CMIP5 models. *Biogeosciences* **10**, 6225-6245, doi:10.5194/bg-10-6225-2013 (2013).

11 Meehl, G. A. *et al.* in *Global Climate Projections* (eds S. Solomon *et al.*) (Cambridge University Press, 2007).

12 Hartigan, J. A. & Hartigan, P. M. The Dip test of unimodality. *Annals of Statistics* **13**, 70-84, doi:10.1214/aos/1176346577 (1985).

13 Moore, J. K., Doney, S. C. & Lindsay, K. Upper ocean ecosystem dynamics and iron cycling in a global three-dimensional model. *Global Biogeochemical Cycles* **18**, GB4028, doi:10.1029/2004gb002220 (2004).

14 Seferian, R. *et al.* Development and evaluation of CNRM Earth system model - CNRM-ESM1. *Geoscientific Model Development* **9**, 1423-1453 (2016).

15 Dunne, J. P. *et al.* GFDL's ESM2 Global Coupled Climate-Carbon Earth System Models. Part I: Physical Formulation and Baseline Simulation Characteristics. *Journal of Climate* **25**, 6646-6665, doi:10.1175/jcli-d-11-00560.1 (2012).

16 Dunne, J. P. *et al.* GFDL's ESM2 Global Coupled Climate-Carbon Earth System Models. Part II: Carbon System Formulation and Baseline Simulation Characteristics. *Journal of Climate* **26**, 2247-2267, doi:10.1175/jcli-d-12-00150.1 (2013).

17 Collins, W. J. *et al.* Development and evaluation of an Earth-System model-HadGEM2. *Geoscientific Model Development* **4**, 1051-1075, doi:10.5194/gmd-4-1051-2011 (2011).

18 Seferian, R. *et al.* Skill assessment of three earth system models with common marine biogeochemistry. *Climate Dynamics* **40**, 2549-2573, doi:10.1007/s00382-012-1362-8 (2013).
19 Ilyina, T. *et al.* Global ocean biogeochemistry model HAMOCC: Model architecture and performance as component of the MPI-Earth system model in different CMIP5 experimental realizations. *Journal of Advances in Modeling Earth Systems* **5**, 287-315, doi:10.1029/2012ms000178 (2013).
20 Tjiputra, J. F. *et al.* Evaluation of the carbon cycle components in the Norwegian Earth System Model (NorESM). *Geoscientific Model Development* **6**, 301-325, doi:10.5194/gmd-6-301-2013 (2013).
21 Locarnini, R. A. *et al.* World Ocean Atlas 2013, Volume 1:Temperature. 40pp (2013).
22 Garcia, H. E. *et al.* World Ocean Atlas 2013, Volume 3: Dissolved Oxygen, Apparent Oxygen Utilisation and Oxygen Saturation. 27pp (2014).
23 Behrenfeld, M. J. & Falkowski, P. G. Photosynthetic rates derived from satellite-based chlorophyll concentration. *Limnology and Oceanography* **42**, 1-20 (1997).
24 Takahashi, T. *et al.* Climatological distributions of pH, pCO2, total CO2, alkalinity and CaCO3 saturation in the global surface ocean. (Oak Ridge National Laboratory, Tennessee, 2014).
